# Generalized Structured Component Analysis in candidate gene association studies: applications and limitations

**DOI:** 10.12688/wellcomeopenres.15396.2

**Published:** 2020-10-08

**Authors:** Paul A. Thompson, Dorothy V. M. Bishop, Else Eising, Simon E. Fisher, Dianne F. Newbury

**Affiliations:** 1 Experimental Psychology, University of Oxford, Anna Watts Building, Radcliffe Observatory Quarter, Woodstock Road, Oxford, OX2 6GG, UK; 2 Max Planck Institute for Psycholinguistics, Wundtlaan 1, Nijmegen, 6525 XD, The Netherlands; 3 Donders Institute for Brain, Cognition and Behaviour, Radboud University, Montessorilaan 3, Nijmegen, 6525 HR, The Netherlands; 4 Department of Biological and Medical Sciences, Oxford Brookes University, Headington Campus, Oxford, OX3 0BP, UK

**Keywords:** genetics, GSCA, Structural equation modelling, simulation, power analysis, developmental language disorder

## Abstract

**Background:** Generalized Structured Component Analysis (GSCA) is a component-based alternative to traditional covariance-based structural equation modelling. This method has previously been applied to test for association between candidate genes and clinical phenotypes, contrasting with traditional genetic association analyses that adopt univariate testing of many individual single nucleotide polymorphisms (SNPs) with correction for multiple testing.

**Methods: **We first evaluate the ability of the GSCA method to replicate two previous findings from a genetics association study of developmental language disorders. We then present the results of a simulation study to test the validity of the GSCA method under more restrictive data conditions, using smaller sample sizes and larger numbers of SNPs than have previously been investigated. Finally, we compare GSCA performance against univariate association analysis conducted using PLINK v1.9.

**Results:** Results from simulations show that power to detect effects depends not just on sample size, but also on the ratio of SNPs with effect to number of SNPs tested within a gene. Inclusion of many SNPs in a model dilutes true effects.

**Conclusions:** We propose that GSCA is a useful method for replication studies, when candidate SNPs have been identified, but should not be used for exploratory analysis.

## Introduction

The earliest discoveries of genetic bases for traits and diseases involved Mendelian traits in which one single genetic variant is sufficient and necessary to cause disease and thus can be directly and consistently correlated to outcome. However, such variants are involved in only a small proportion of disease and are rare within populations. The majority of common diseases, disorders and traits are usually instead described in terms of the Common Variant Common Disease (CVCD) model ( Cargill *et al*., 1999), where a number of common genetic variants have small effects, and only assume importance when combined with other genetic or environmental factors: so-called complex multifactorial aetiology.

Identifying these small effects is difficult. Early studies assumed the involvement of a moderate number of common variants and employed single-variant linkage and association methods to identify contributory factors ( Lander, 1996). However, there is a growing awareness that the variants that influence complex disorders are numerous and have minuscule effects ( Gibson, 2012; Visscher *et al.*, 2012). Accordingly, newer analysis models attempt to integrate variation across markers and interactions between them ( Cordell, 2009). Nonetheless, these methods are far from straightforward as they need to be able to effectively consider variants across a range of allele frequencies and effect sizes. One problem confronting the researcher is that of multiple testing: the options are either to focus attention on a few variants within candidate genes, and run the risk of missing effects of importance elsewhere on the genome, or to look at hundreds of thousands of variants, in which case enormous sample sizes are needed to identify true effects of minute sizes among a potential plethora of false positives. Even with very large samples, small effects may go undetected because stringent corrections for multiple testing have to be used. Furthermore, large sample sizes are difficult to attain if the phenotype of interest is a condition or trait that can only be detected via individual testing, and which is not routinely assessed. This is the case for the condition that is the main focus of our research, namely Developmental Language Disorder – previously known as Specific Language Impairment, (SLI; see Bishop *et al.*, 2017). Here we evaluate an alternative approach to identifying genetic influences, which does not look for statistical associations with individual single nucleotide polymorphisms (SNPs), but rather selects SNPs that may be regarded as indicators of a common genetic effect, and tests for association of a clinical pathway with the combined genetic effect. Similarly to Romdhani *et al*., (2015), we refer to ‘clinical pathways’ as the weighted sum of phenotypes.

Generalized Structured Component Analysis (GSCA) was introduced by Hwang & Takane (2004). The method has some superficial similarities to structural equation modelling (SEM), which typically follows a covariance-based approach and requires substantial sample size to ensure adequate model fit. However, a key difference is that GSCA uses a weighted sum as the basis for ‘factors’ or ‘traits’. The comparison of the two methods can be likened to using a principal components analysis (GSCA) instead of factor analysis (SEM). A key benefit of the GSCA method is that it can achieve satisfactory model fit even when with relatively small samples ( Chin & Newstead, 1999; Tenenhaus *et al.*, 2005). This is possible because it uses a parameter estimation method called alternating least squares ( Hwang & Takane, 2004). In this method, the model is not fitted using the entire covariance matrix at once; rather, the models for individual ‘factors’ (genes and phenotypes in our case) are fitted alternately to the covariance model for the latent ‘factors’ (structural model).

Romdhani *et al.*, (2015) first proposed using GSCA for genetic association analysis with candidate genes. They reported a simulation study with a relatively small number of SNPs and sample sizes that, while large by standards of statistical model-fitting, were relatively small for genetic associations (N=1000). The results showed good support for the applicability of the method under those conditions. They also presented an example application using the Quebec Child and Adolescent Health and Social Survey (1999) data ( Paradis *et al*., 2003).

Our interest in the GSCA approach arose because we were studying genetic influences on neurodevelopment in children with an additional X or Y chromosome. Obtaining large samples for this population is problematic, so finding an appropriate analysis to consider multiple variants was challenging. We tested a model that assumed that the effect of common genetic variants on neurodevelopment would be enhanced in this group (estimated effect size of *d* = 0.5), because of interactions with the effect of the additional sex chromosome. Using GSCA, we failed to find any effect on the phenotype for SNPs in two candidate genes that were selected on functional grounds ( Newbury *et al.*, 2018). Because our simulation studies showed that with GSCA, we were adequately powered to detect an effect size (d) of 0.5 with a sample of 130 cases, we could conclude that our failure to find an effect reflected a genuine null finding, rather than being due to inadequate power.

Our experience with the method led us here to explore further its potential with small samples. We first conducted a replication of existing findings of association from the literature on developmental language disorder using the GSCA method with an existing dataset, the SLIC dataset ( SLI Consortium, 2002). We compared the findings of the GSCA with conventional single SNP analysis, for four groups of SNPs, identified on the basis of prior literature. The first two groups were SNPs located on genes that had previously been shown to be associated with quantitative speech and language phenotypes in the full SLIC data set (Study Part 1). Here we predicted that GSCA should confirm the association. These two groups were contrasted with SNPs from two other genes that acted as negative controls, in that we predicted they would not show association with DLD phenotypes.

Given the promising results of this exercise, we then conducted an extended range of simulations, using smaller sample sizes and larger numbers of SNPs than Romdhani *et al.* (2015). Furthermore, we compared our findings with simulations using both PLINK and Romdhani *et al.* (2015) methods for comparison (Study Part 2).

## Methods

### Part 1: Comparison of GSCA vs conventional association analysis with an existing dataset

***SLIC dataset.*** The SLIC cohort has previously been reported in multiple earlier studies ( Falcaro *et al*., 2008; Gialluisi *et al*., 2014; Monaco, 2007; Newbury *et al*., 2009; Newbury *et al*., 2011; Nudel *et al*., 2014; Simpson *et al*., 2015; SLI Consortium, 2002; SLI Consortium, 2004); the following description is based on Simpson *et al.* (2015). The sample consisted of British nuclear families that included at least one child affected by SLI, defined as expressive and/or receptive language skills (ELS and RLS, respectively, measured on the Clinical Evaluations of Language Fundamentals-Revised; Semel *et al.*, 1987) at least 1.5 SD below the normative mean and non-verbal IQ at least 78 (i.e. not more than 1.5 SD below that expected for their age). Non-verbal skills were measured by the WISC Perceptual Organisation Index, a composite score derived from Picture Completion, Picture Arrangement, Block Design and Object Assembly subtests of the Wechsler Intelligence Scale for Children - III ( Wechsler, 1992). Non-word repetition (NWR) was a 28-item verbal repetition of orally presented nonsense words. DNA was extracted from peripheral blood or buccal smears and all samples were genotyped on the Illumina HumanOmniExpress-12v1 Beadchip (San Diego, CA, USA).

All SLIC genotype data had previously undergone quality control using standard procedures, as described in Nudel *et al.* (2014): SNPs and samples with a genotype success rate below 95% and/or heterozygosity rates ±2 SD from the mean were removed, as were all SNPs with a minor allele frequency of less than 1% or a Gentrain (genotype quality) score below 0.5. Inheritance data within families were used to exclude SNPs and samples with an error rate of above 1%. Control data (HapMap release #3) were employed through a principal component analysis to exclude individuals with divergent ancestry, and samples with gross chromosome rearrangements or discordant sex information were removed. After filtering, 954 out of 1047 individuals and 618,879 out of 727,913 genomewide SNPs remained before imputation.

***Subsample selection.*** To evaluate the sensitivity of the GSCA method in a small sample, we selected a subset of 125 individuals from the SLIC cohort (subsequently, four cases were removed due to missing data in the imputed SNPs, hence all figures reported for GSCA will be based on N=121), corresponding to the set of cases described by Simpson *et al.* (2015).

These were all probands and unrelated to one another. These individuals were all affected with language disorder and represented a case cohort of the kind typically ascertained for genetic analyses, but as shown in Table 1, there was a good spread of scores in the three phenotype measures.

**Table 1.** Age-scaled scores for the three phenotype measures.

Phenotype	n	mean	sd	min	max	skew	kurtosis
Expressive language	121	68.19	10.49	50	98	0.41	0.35
Nonword repetition	121	81.09	19.76	55	136	0.56	-0.28
Receptive language	121	78.07	13.64	50	131	0.36	1.06

***Candidate gene selection.*** Four candidate genes were examined; two of these, *CNTNAP2*, a single linkage disequilibrium (LD) block corresponding to the most robustly associated region, and *ATP2C2* were selected because they have previously been shown to be associated within the full SLIC cohort ( Newbury *et al*., 2009; Vernes *et al*., 2008). The remaining two genes had not previously been shown to be associated with language phenotypes: *FOXP2* was selected as a control gene because although rare, highly penetrant mutations in this gene cause a severe developmental speech and language disorder (see Graham & Fisher (2015) for review), no association with common variants has been found in the SLIC sample ( Newbury *et al*., 2002). The fourth gene, ( *UBR1*), was selected as a control for false positives on the basis that it emerged as the maximally-associated gene within the dataset in hand (125 independent cases) on genome-wide analysis with PLINK. As shown in Table A4 (see *Extended data*; Thompson *et al.*, 2019), four of the seven SNPs in this gene were associated with p < 0.05 with at least one of the three language phenotypes - *UBR1* has not previously shown any association with language phenotypes in the full SLIC sample, and these associations do not achieve genome-wide significance. There is no functional reason to suppose this is a meaningful finding, so inclusion of this gene provides a stringent test of the sensitivity of GSCA to generate type I errors.

**Table 2.** Candidate gene position in relation to two human genome references ( hg18 and hg19). These are the positions for the entire genes, not regions of interest in the current study.

Gene	hg18	hg19
CNTNAP2 (NM_014141)	chr7:145,444,386-147,749,019	chr7:145,813,453-148,118,086
FOXP2 (NM_148898)	chr7:113,842,288-114,118,328	chr7:114,055,052-114,331,092
ATP2C2 (NM_014861)	chr16:82,959,634-83,055,294	chr16:84,402,133-84,497,793
UBR1 (NM_174916)	chr15:41,022,390-41,185,578	chr15:43,235,098-43,398,286

***SNP selection.*** Genotypes across the regions described were extracted from the SNP genotype data within PLINK v1.9 ( Purcell *et al*., 2007). *CNTNAP2* and *ATP2C2* are large genes containing many SNPs; therefore, we filtered these two genes to include only those regions of association that had been previously published. 

The complete *CNTNAP2* gene contained 554 directly genotyped variants and was filtered to include the region of association as described in previous literature, chr7:147126276-147232255 (hg18, RefSeq assembly accession number GCF_000001405.12). The complete *ATP2C2* gene contained 82 directly genotyped variants and was filtered to include the region of association as described in previous literature, chr16:83005506 - 83022754 ( hg18). The *FOXP2* gene only contained 13 directly genotyped SNPs, and the *UBR1* gene contained 15 SNPs, so no pruning was required for these genes.

The dataset was then further filtered to include only SNPs with minor allele frequency (MAF) greater than 0.03, and pairwise LD values smaller than 0.8. This gave a dataset of 61 SNPs (23 *CNTNAP2*, 18 *ATP2C2*, 13 *FOXP2*, 7 *UBR1*).

***Imputation.*** One question about GSCA is whether it would perform better with a larger number of SNPs. In human genetics, imputation is commonly used to increase the density of SNPs in a region by statistically inferring unobserved SNPs. Genome-wide imputations were completed to derive a fuller representation of variation across the genes of interest, restricted to the same regions of interest defined above for *CNTNAP2* and *ATP2C2*, and for the whole gene for *FOXP2* and *UBR1*. After filtering out SNPs with minor allele frequency < 0.01, call rate < 0.98, Hardy Weinberg p < 1.0 × 10 ^-6^, and individuals with call rate < 0.98 and non-European ancestry, the genotype data were imputed on the Michigan Imputation Server ( Das *et al.*, 2016). Samples were phased using Eagle v2.3 and imputed against the HRC r1.1 panel (EGA accession number EGAD00001002729), which includes genotypes for 32,470 samples across 3,9635,008 variants. After filtering on imputation quality (Rsq≥0.7) and allele frequency (MAF>0.03) and pruning for SNPs in high linkage disequilibrium (R2>0.8), the imputed dataset included 288 SNPs (FOXP2, N=99; CNTNAP2, N=86; UBR1, N=36; ATP2C2, N=67; 17 of these SNPs were from the original hard-called genotyped data).

***Generalized structured component analysis.*** We briefly restate the technical details of the GSCA method: Comprehensive method specifications can be found in Hwang & Takane (2004) and Romdhani *et al.* (2015). Suppose we have both observed candidate SNPs and phenotypes that we denote ( *X*_1_, ..., *X _J_*, *X _J_*_+1_, ..., *X _K_*), where there are *J* SNPs and *K* phenotypes. As with any other SEM model, we map manifest variables onto unobserved latent variables (more specifically, the weighted sums of SNPs or phenotypes). We define the *l ^th^* latent variable by *γ _l_* = ∑ *_i∈S _l__* *w _il_ X _i_*, *l* = 1,..., *L*., where *S _l_* denotes the set of indices of observed candidate SNPs and phenotypes mapped to the *l _th_* latent variable, and *w _il_* is the weight for each observed variable in that latent variable. In this model framework, the *J* SNPs map to *G* genes and similarly, *K* phenotypes map to a single clinical pathway, denoted respectively by ( *γ*_1_,..., *γ _G_*, *γ _DLD_*).

The structural part of the model is similar to the structure used in SEM. The latent variable relationships can be specified as follows,

$$\gamma_{l}=\sum_{l\prime =1,l\prime \neq l}b_{l\prime l}\gamma_{l\prime }+\epsilon_{l},\;l=1,\ldots ,L.$$

where *∈ _l_* denotes the model error term, and *b _l'l_* is the path coefficient, which links between latent variables *l'* and *l*. In our examples, the path coefficient is the link between gene(s) and a clinical pathway. Unlike structural equation modelling (SEM), GSCA does not necessarily require univariate normality to ensure multivariate normality; however, multivariate normality remains an important requirement in GSCA models. This is another beneficial feature of the GSCA approach over a standard SEM equivalent.

The statistical software R (version 3.6.0 2019-04-26; R Core Team, 2019) and R package ASGSCA ( Romdhani *et al.*, 2019) was used to fit the GSCA models in both simulations and SLIC datasets.

**Figure 1.**
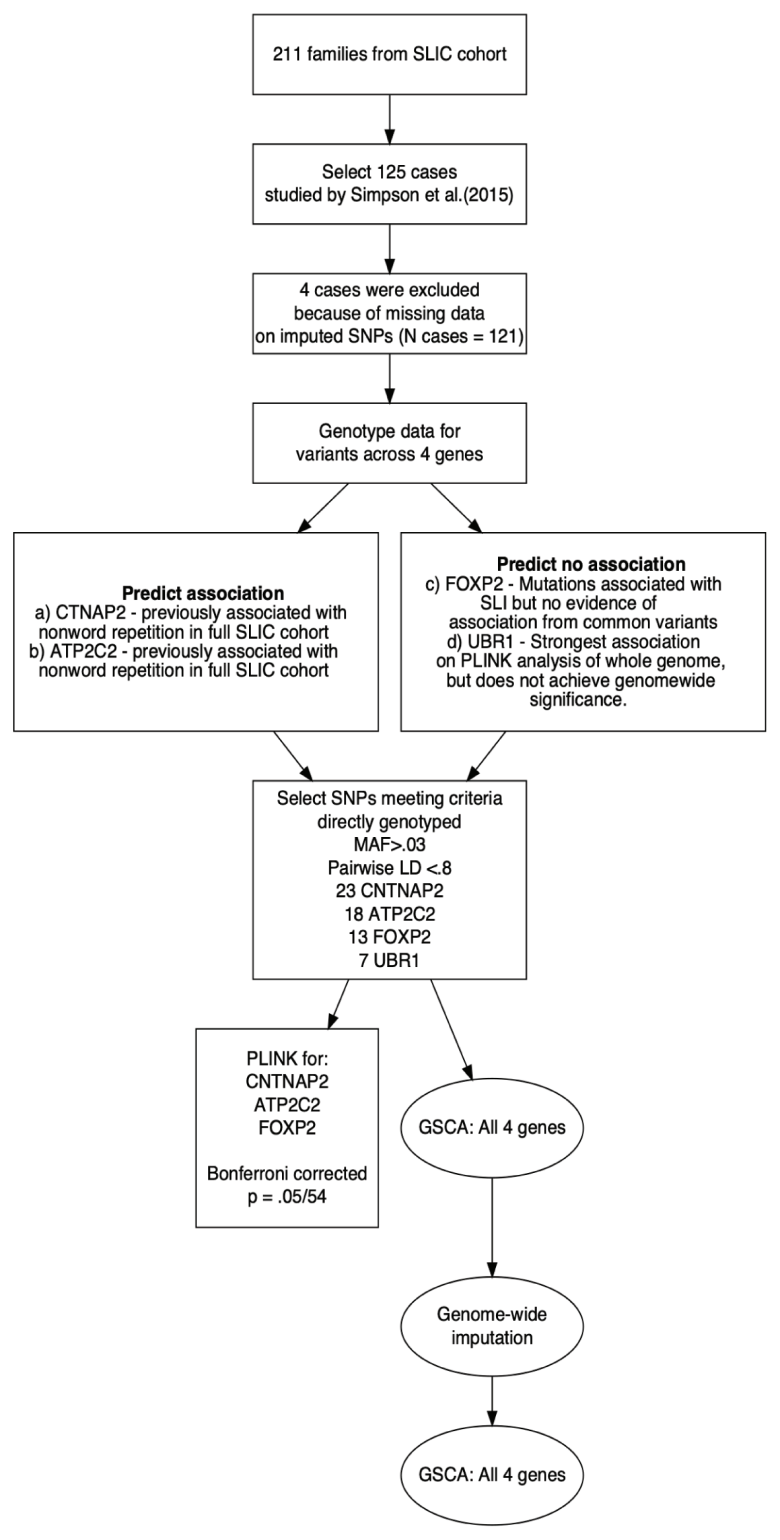
Flow diagram of SLIC cohort analyses (Study Part 1). GSCA, Generalized Structured Component Analysis; LD, linkage disequilibrium; MAF, minor allele frequency; SLIC, Specific Language Impairment Consortium; SNPs, single nucleotide polymorphisms.

***Part 1 analysis overview.*** Figure 1 summarizes the workflow for the comparison between single-SNP association analysis and GSCA.

### Part 2: Performance of GSCA with simulated data

We simulated a range of scenarios to determine the applicability and limitations of the GSCA method in genetic association analysis in candidate genes (54 combinations of five variables). The simulations varied the following factors: (1) number of SNPs; (2) number of SNPs with an effect on phenotype; (3) number of participants; (4) phenotype correlation; and (5) effect size, quantified as size of correlation between SNPs exhibiting an effect and phenotypes. We conducted simulations with effect sizes of r = 0.1, 0.15, and 0.2.

In addition, we contrasted situations where the SNPs with an effect were clustered within a gene, or spread across genes. This extends the scope of simulations presented in Romdhani *et al.* (2015), although we do not investigate more complex path arrangements as they did, such as cross loadings, multiple paths from one gene, or traits that load onto more than one phenotype. Their simulations did not consider sample sizes less than 1000, and typically only looked at genes with one or two SNPs mapped to them. Here, we assessed the performance of GSCA using smaller sample sizes (N=100). Subsequently we investigated N=500 and N=1000 with the PLINK simulation, but the sample size of 100 was sufficient to achieve adequate power >80% for the vast majority of simulations using GSCA. We considered how well the method performs with data that are more sparse and with more SNPs per gene. Due to the computational and time constraints of running such a large number of combinations, we used 100 replications per combination, which is less than Romdhani *et al.* (2015), who used 1000 replications per combination, having found this generally adequate to illustrate how different parameter variations affect power.

**Figure 2.**
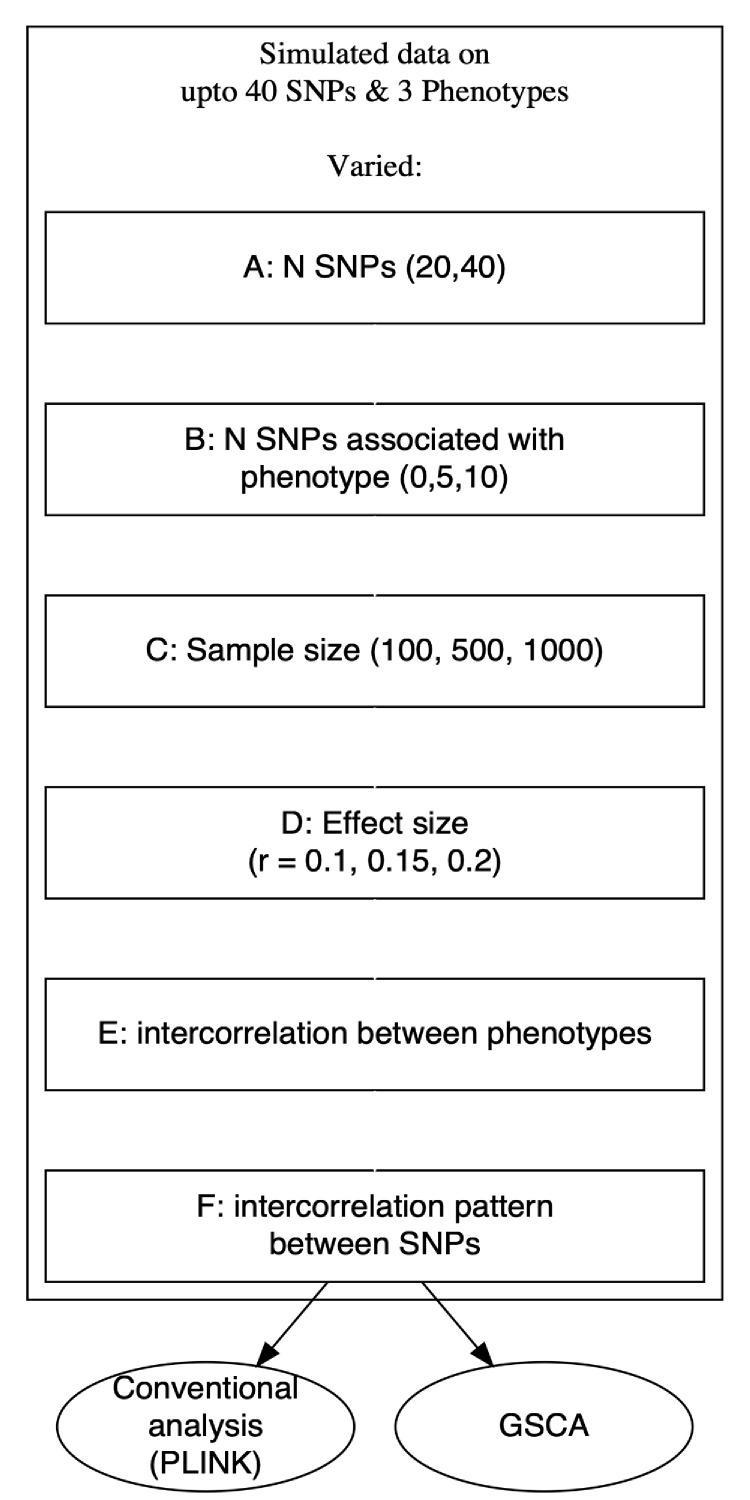
Flow diagram of simulations (Study Part 2). GSCA, Generalized Structured Component Analysis; SNPs, single nucleotide polymorphisms.

***Assessment of power.*** Beyond simple modelling and hypothesis testing procedures, power for multivariate analyses can be complex to quantify. The most convenient and accurate method, particularly in SEM, is to use Monte Carlo simulation ( Wolf *et al*., 2013). The Monte Carlo method simulates multiple data sets from the same model conditions and then fits the same model for each simulated data set. This provides an assessment of parameter estimate bias, standard error bias, and coverage ( Muthén & Muthén, 2009). Following this approach, a range of parameter combinations are simulated to determine the effect on statistical power, as shown in Figure 2.

**Figure 3.**
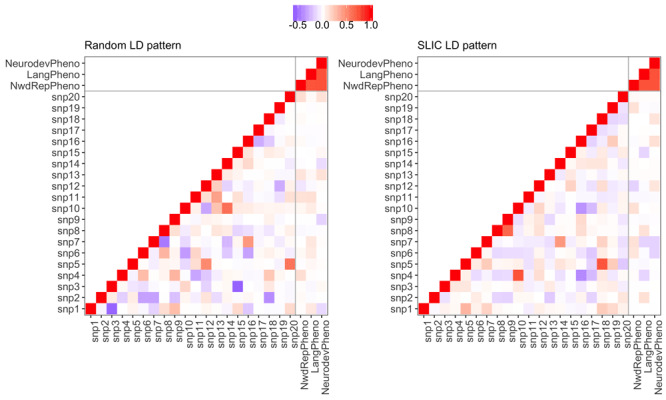
Example heatmaps of the pattern of correlations among 20 SNPs and three phenotypes, in the case where there are five SNPs with an effect on the phenotype. Left: random correlation pattern; Right: randomly sampled correlation pattern from SLIC data. LD, linkage disequilibrium; SLIC, Specific Language Impairment Consortium; SNPs, single nucleotide polymorphisms.

The simulations were devised to reflect linkage disequilibrium, with the degree of correlation between SNPs in a gene to mimic a real data set (SLIC data). This was achieved by either sampling from the SLIC data correlation matrix directly to form a structure to simulate from, or generating a random pattern that had the same a representative frequency of magnitudes of correlations in the matrix. In addition, the three measures of language phenotype were simulated to be intercorrelated. The data were simulated simply as the correlation between SNPs and phenotypes directly without formal model structure. Figure 3 shows the heatmaps of the pattern of correlations among the SNPs and phenotypes for two situations: (a) with SNP correlation structure as a random pattern ( Figure 3, left) or (b) with SNP correlations pattern sampled from the SLIC data ( Figure 3, right), where association effects between phenotypes and SNPs were allowed to be randomly allocated across the gene and different SNPs could be associated to different phenotypes.

The simulations were devised to match real data as closely as possible to assess the performance of GSCA against PLINK single SNP analyses. The script to run the simulations is provided (see *Software availability*; Bishop & Thompson, 2020).

## Results

### Part 1: Comparison of GSCA vs conventional association analysis with an existing dataset

In part 1 of the study, we used a subset of data from the SLIC cohort to check first whether associations that had been found in the analysis of family data from the full cohort would be detectable in this subset of individuals despite the reduced statistical power and second, whether GSCA is less prone to identify spurious associations (type I error) than more conventional univariate association analysis conducted using PLINK v1.9 ( Purcell *et al*., 2007).

***SLIC data: association testing with directly observed SNP set.*** Conventional association analysis of the SLIC sample set was first conducted directly genotyped SNPs in *CNTNAP2, ATP2C2 and FOXP2*. All 54 SNPs were tested for association against the three language phenotypes: expressive and receptive language, and nonword repetition. Analyses were performed with PLINK 1.9 using a quantitative model of association ( Purcell *et al*., 2007), and a Bonferroni-corrected significance level of 0.05/54=0.0009 - even without a more stringent correction taking into account the fact that there are three measures of the phenotype. A full summary of the results is provided in Table A2 ( *Extended data*; Thompson *et al.*, 2019). There were no significant associations for any of the three phenotypes with SNPs from *CNTNAP2*, *ATP2C2* or *FOXP2*.

As noted above, *UBR1* had been selected for study because it had the strongest evidence of association with language phenotypes on whole genome testing (over 600,000 SNPs from around 20,000 genes). Table 3 shows those SNPs in *UBR1* with p-values less than 0.05. One SNP had p < 0.05 for Receptive and Expressive Language, and four SNPs met this criterion for Nonword Repetition. Note, however, because this gene had been selected from analysis of a much larger pool of genes on the basis of having the strongest association, these p-values cannot be meaningfully interpreted. *UBR1* is included as a control gene that allows us to compare findings from PLINK and GSCA analyses.

**Table 3.** UBR1 SNPs with nominally significant association to language phenotypes.

CHR	SNP	Phenotype	*p*
15	rs3759792	NWR	0.001437
15	rs17719808	ELS	0.021860
15	rs17719808	RLS	0.000451
15	rs3736054	NWR	0.000678
15	rs2412752	NWR	0.000009
15	rs16957385	NWR	0.000036

CHR, chromosome; ELS, expressive language skills; NWR, non-word repetition; RLS, receptive language skills; SNP, single nucleotide polymorphism.

***GSCA approach for pathway-based association.*** The GSCA method was used to re-analyse data from the same 121 probands following the approach described in Romdhani *et al.* (2015). Note that this method does not require correction for the number of SNPs, but correction for the number of structural paths is needed to maintain the desired alpha level for individual paths (i.e. links between genes and clinical pathways). Thus, we adopted an alpha level of 0.05/4=0.0125.

**Figure 4.**
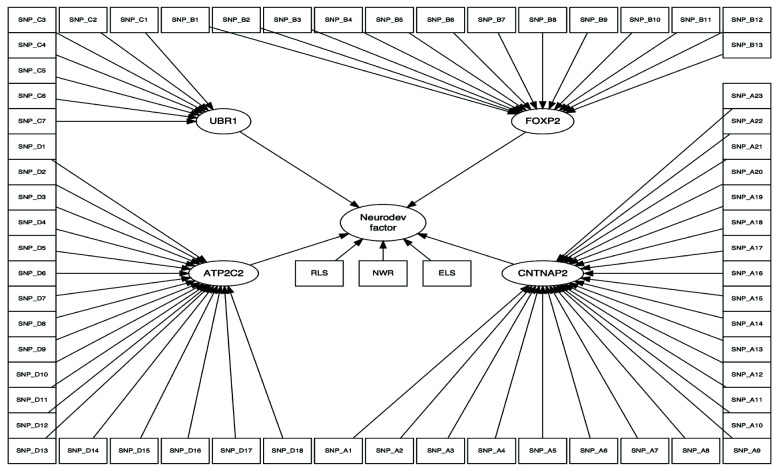
Path diagram for SLIC data. ELS, expressive language skills; NWR, non-word repetition; RLS, receptive language skills.

Figure 4 shows the GSCA path diagram with structural relationships between latent variables, and the weighted sums of SNPs or phenotypes which feed into their respective factors (representing candidate genes: *CNTNAP2*, *FOXP2*, *ATP2C2*, and *UBR1*, or the clinical pathway: developmental language disorder). Square shapes indicate measured variables (SNPs or phenotypes), and ellipse shapes are latent variables.

Table 4 reports the results of the GSCA association analysis. Individual weights are not interpretable in a comparable way to SEM path loadings as the GSCA weights represent the joint effect of all SNPs (within the gene) mapped to the gene on all phenotypes mapped to the clinical pathway ( Romdhani *et al*., 2015). The two associations reported in Vernes *et al.* (2008) and Newbury *et al.* (2009) between the SNPs in *CNTNAP2* and *ATP2C2* genes and the language factor were replicated in this smaller subset of the original data. In addition, the null finding of no association between *FOXP2* and any language phenotype was also replicated ( Newbury *et al*., 2002). Furthermore, the association between *UBR1* and any language phenotype within this subset of data was not replicated using the GSCA method. *ATP2C2* and *CNTNAP2* are quite robust associations, while the *UBR1* result is consistent with our interpretation of a false positive finding from the PLINK analysis.

**Table 4.** Path estimates and P values for Generalized Structured Component Analysis on Specific Language Impairment Consortium data. P values are obtained by permutation testing.

Gene	Language phenotype	*p*
FOXP2	0.382	0.560
CNTNAP2	-1.357	<0.001
UBR1	0.370	0.260
ATP2C2	-0.832	<0.001

***GSCA analysis on the SLIC data following imputation.*** The focused analysis of 61 SNPs suggested GSCA was not only more powerful than conventional regression analysis of individual SNPs for detecting association in small samples, but also more valid in avoiding type I error. This raised the question of whether performance of GSCA could be enhanced by adding further information by imputing values for additional SNPs. To address this question, we repeated the GSCA analysis using the SLIC data after an imputation process had been applied substantially increasing the number of available SNPs included in the analysis (after pruning for MAF, imputation quality and LD, Total SNPs=288; *FOXP2*, N=99; *CNTNAP2*, N=86; *UBR1*, N=36; *ATP2C2*, N=67). We then filtered the data further by removing perfectly correlated SNPs, as the GSCA method has no mechanism to deal with collinearity. The final numbers of SNPs were as follows: total SNPs=244; *FOXP2*, N=85; *CNTNAP2*, N=60; *UBR1*, N=32; *ATP2C2*, N=67. The number of individuals was also matched so that the same individuals were found in both samples giving N=121.

Although we might have anticipated that adding information via imputation should improve performance of GSCA, as shown in Table 5, it had the opposite effect. Specifically, we saw no significant paths when the analysis was run on 244 SNPs. We suspected this reflected the fact that weights from individual SNPs become diluted when a large number of SNPs are included in the analysis, and we therefore conducted simulations to investigate this possibility.

**Table 5.** Path estimates and P values for Generalized Structured Component Analysis on imputed Specific Language Impairment Consortium data.

Gene	Language	*p*
FOXP2	0.553	0.462
CNTNAP2	-0.385	0.768
UBR1	-0.649	0.692
ATP2C2	0.284	0.356

### Part 2: Performance of GSCA with simulated data

Our analysis of empirical data suggested that GSCA performed well with small samples and small numbers of SNPs, showing good sensitivity to detect true associations and avoidance of type I error, and outperforming conventional association analysis of individual SNPs and phenotypes. A surprising finding, however, was that performance broke down when the number of SNPs was increased by using imputed values. This led us to explore the conditions under which GSCA performance was optimal. Our main focus was on the question of how well GSCA could detect true associations in simulated data, i.e. statistical power of the method.

**Figure 5.**
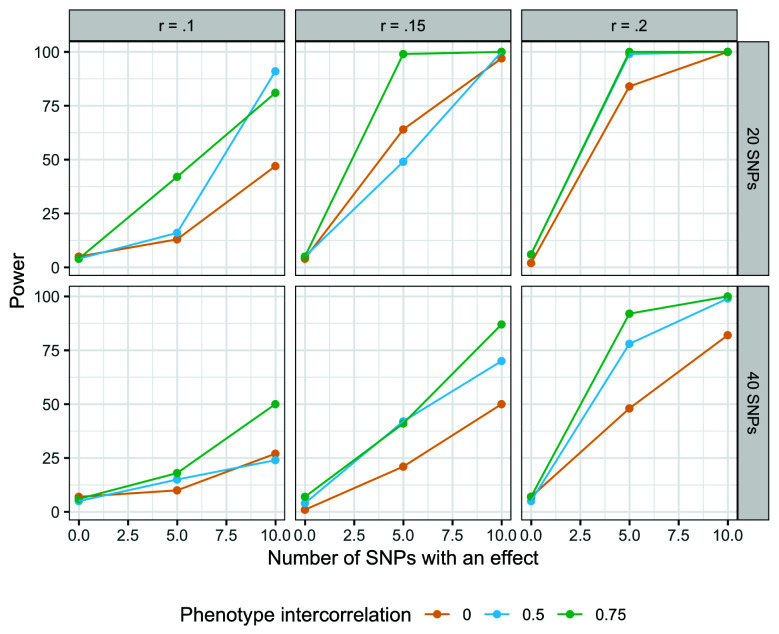
Power curves displaying results of GSCA simulations (random pattern of SNP LD with effect). GSCA, Generalized Structured Component Analysis; LD, linkage disequilibrium; SNP, single nucleotide polymorphism.

Figure 5 summarizes the results of the simulation study using random pattern of SNP’s LD exhibiting an effect when varying sample size, number of SNPs, number of SNPs having an effect on a phenotype, and size of effect (correlation). The top row of plots are the total number of SNPs in the gene, and the columns represent the size of effect between individual SNPs with an effect and particular phenotype. The colour of each line indicates the correlation between phenotypes. We show only results where N=100. When N was 500 or more, power was close to 100% for all conditions.

Consider the first row of plots in Figure 5, which shows the situation when we simulate a smaller number of SNPs (20). We can see that the power to detect an effect increases as we would expect when the effect size increases. A further point to note is that false positive rate with this method is very low: thus, when the number of SNPs with an effect is zero, it is very rare to find an effect that achieves statistical significance. The increase in effect size (i.e. correlation between phenotype and SNP), seen in the plots from left to right panes, is in line with the corresponding increase in power.

As the effect size increases (moving left to right across columns), the power increases to more reasonable levels (>80%). The differences in power when the effect size is larger is influenced by sample size or correlation among phenotypes. Lower correlations between phenotypes tend to reduce power of the GSCA analysis, which is consistent with findings from Romdhani *et al.* (2015). A more unexpected finding was that power increased with the proportion of associated SNPs among all SNPs. This proportion is controlled by the total number of SNPs, the number of genes (due to the weights estimated per factor - gene or clinical pathway in this case), and the number of significant SNPs in total. If the ratio is low, true effects get swamped. The reduction in power between curves can be seen in Figure 5: differences between the top row (total number of SNPs = 20) and the bottom row (total number of SNPs = 40) can be attributed to the relative proportions of SNPs with effect among all the SNPs. In the top row, we have a higher proportion of SNPs with an effect, so GSCA is able to detect a true effect more consistently. This pattern is consistent with our analysis of SLIC data, where increasing the number of SNPs from 61 to 288 eliminated the effect of interest.

Sufficient power at 80% is reached when either the effect size is typically larger than 0.1 and the proportion of SNPs with an effect is larger than 25% (if effect size is 0.15, this is only seen when there are higher correlations between phenotypes). In almost all cases, simulations with zero correlation between phenotypes did not achieve sufficient power (>80%).

**Figure 6.**
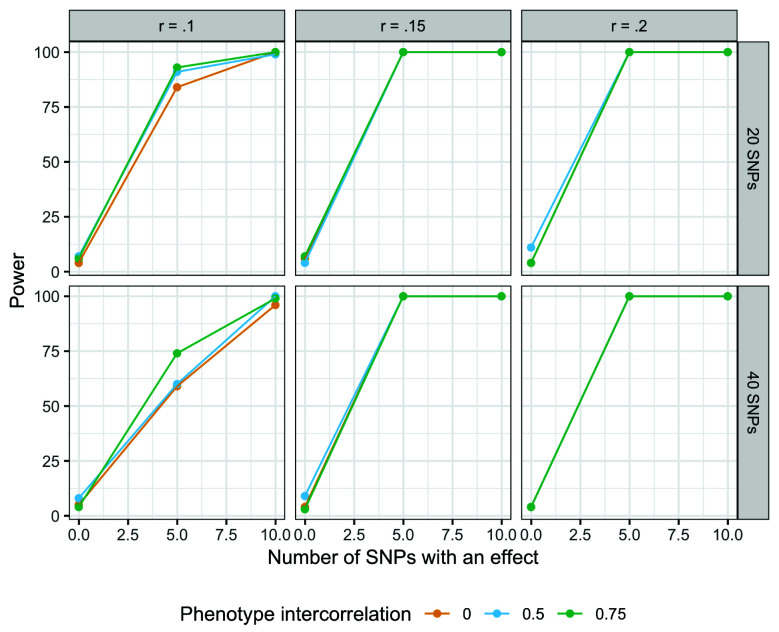
Power curves displaying results of GSCA simulations (random SLIC data pattern of SNP LD with effect). GSCA, Generalized Structured Component Analysis; LD, linkage disequilibrium; SLIC, Specific Language Impairment Consortium; SNP, single nucleotide polymorphism.

Turning to Figure 6, in this simulation SNPs with an effect occur at random, but the LD pattern is sampled from the original SLIC data correlation matrix. Once again, we find that the false positive rate is typically low (below 5%) for all simulations. In almost all simulations, with the exception of the lower left panel, the GSCA analysis appears to be well powered (>80%) given that at least five SNPs are present with an effect, regardless of total number of SNPs (N=20 or 40) or magnitude of phenotype correlation. The apparent differences between the random and SLIC-based simulations are likely as a result of the LD structure in the SLIC data has high concentrations of medium to large SNP LDs than the random structure simulations.

In this simulation, we confirm that sample size, size of effect, and ratio of SNPs with an effect on phenotype per gene remain the most influential factors for the analysis. Smaller effect size reduces the power achieved when there are fewer SNPs with an effect and the reduction is also co-dependent on the total number of SNPs, effectively watering-down the power to detect any effect.

***Alternative approach simulations: single SNP analysis (PLINK).*** The traditional approach to association analysis is to conduct a linear regression analysis for each SNP and phenotype, using a multiple testing correction. This correction is often Bonferroni but the PLINK ( Purcell *et al.*, 2007) software offers a range of alternative correction options as standard (Holm, SIDAK and False Detection Rate control - with either Benjamini-Hochberg or Benjamini-Yekutieli; https://zzz.bwh.harvard.edu/plink/anal.shtml#adjust). 

This method requires large sample sizes to have adequate power to detect small effects, compounded by the stringent correction to alpha levels that is required when many SNPs and/or phenotypes are considered. We compared the power of the PLINK approach using FDR control (Benjamini-Hochberg) with GSCA for datasets simulated using the same methods. We treated a run of the simulation as having a positive result if any p-value for any SNP/phenotype combination fell below the corrected alpha level after adjusting the FDR rate.

**Figure 7.**
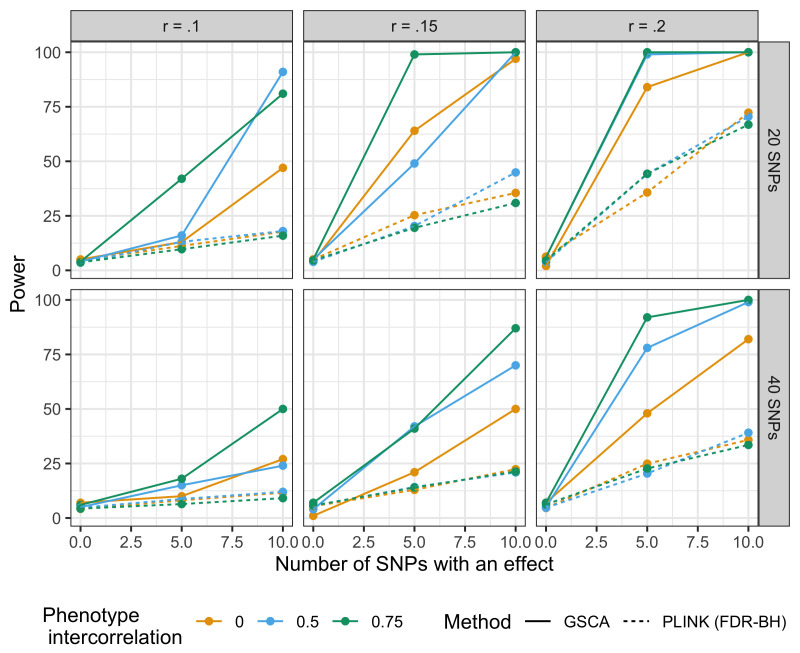
Plots displaying results of PLINK analysis and GSCA for simulated data to compare the relative statistical power achieved under different conditions. GSCA, Generalized Structured Component Analysis; SNP, single nucleotide polymorphism.

The results in Figure 7 show results for a sample of 100 subjects simulated with a random LD correlation pattern, varying by effect size (top ribbon), number of SNPs (right ribbon), and number of SNPs with an effect (x axis). The continuous lines show the original GSCA results, and the dotted lines show the PLINK results. Different correlations between phenotypes are shown as different colour curves. Because the analysis is considerably faster than GSCA, we used 1000 runs of the simulation for PLINK simulations. As expected, the false positive rate for PLINK was around 5% when the FDR control was applied to adjust for number of SNPs and number of phenotypes. GSCA achieved a comparable false positive rate when no effect was present but higher statistical power at all effect sizes and sample sizes. PLINK was generally not well powered to find true effects if sample size was less than 500. Sample size was increased in the PLINK simulations as the simulations under N = 100 did not reach a typical power threshold of 80%.

## Discussion

We present results from an applied example using the SLIC data to show the validity of the GSCA method to replicate previously reported findings and the validity of our simulations within a real data set. In addition, we included a simulation study for candidate gene association studies using the GSCA method to investigate how power and false-positive rates are affected by effect size, sample size and number of SNPs.

In targeted GSCA analyses, we reproduced in a subset of the original data the main findings of the studies by Vernes *et al.* (2008) and Newbury *et al.* (2009), in which the *CNTNAP2* and *ATP2C2* genes were associated with quantitative measures of language ability in individuals affected by language disorder. These findings indicate that the GSCA method is sensitive for detecting complex genetic associations.

In contrast, GSCA did not detect association to *FOXP2* or *UBR1*. These findings again align with that expected from previous analyses. Although the *FOXP2* gene is a robust candidate for language disorder, such cases usually involved monogenic mechanisms and rare mutations ( Graham & Fisher, 2015; Morgan *et al.*, 2016) and these would not have been detected by the screen employed here, which included only common variations (MAF>0.03). Previous studies have failed to detect association between common *FOXP2* variation and language measures in individuals affected by developmental language disorder ( Newbury *et al*., 2002). The null findings with *UBR1* are particularly illuminating, given the pattern of association found on PLINK ( Table 3). There are two key differences between the analysis methods. The first is that GSCA makes use of structure in the data, taking into account inter-correlations between the different phenotypes representing a phenotypic path. Although individual SNPs appeared associated with a specific language phenotype in the PLINK analysis, this would not contribute to a significant path being observed if, for instance, the correlation with another language phenotype was of opposite sign. In addition, even when SNP samples are filtered for linkage disequilibrium, there may be some degree of inter-correlation between SNPs within a gene, and this information will also contribute to the pattern of association between pathways and genes. The GSCA would also potentially be sensitive to the situation when a gene contained a range of SNPs that were not in linkage disequilibrium, but whose variants affected gene function in similar ways, leading to a common genetic pathways affecting the phenotype.

The second difference between methods is that univariate association analysis of individual SNPs and phenotypes requires stringent correction to be imposed to avoid false positives from multiple analyses. The correction needed with GSCA is milder, because it is based on the number of paths in the analysis, not the number of SNPs and phenotypes.

When we repeated the GSCA analysis using an imputed version of the SLIC data that included an increased number of variants, the previously significant associations were no longer found. This finding motivated the decision to conduct the simulation study. The imputation methods followed standard procedures used routinely in single SNP genetic analyses, so it seems unlikely that the imputation method was the source of difference. The only other possibilities were that the addition of more SNPs gave rise to the change in results. Simulation provided a means to test the limitations of the method under different scenarios, so that we could explain the difference and rule out a false positive result.

The first simulation study varied a range of potential parameters to test the GSCA method under different theoretical scenarios. We limited the simulations to a small number of phenotype correlation magnitudes, effect sizes, and only simple structural models. In addition, we explored two possible patterns of SNPs LD correlations, random and randomly sampled from the SLIC data correlation matrix across the pool of candidate SNPs from the set of selected genes. Each model was permuted 100 times for each simulation scenario data set, so that the results of the simulations were robust.

As could be expected, reducing the effect size in the SNPs exhibiting an effect has a dramatic effect on the power across all combinations of simulation parameters. A less obvious finding was that the ratio of the number of SNPs with an effect relative to the total SNPs per gene affected the power. When weights are estimated for the paths from SNPs to the latent variable, the size of individual weights decreases as the number of weights increases. Thus, when the total number of SNPs per gene is large, more SNPs with an effect are needed to maintain a low false positive rate. With a larger number of SNPs contributing weights to the sum, the size of these weights is proportionally smaller and more difficult to estimate, as there is more uncertainty in the sign of the weight and whether it is significantly different from zero. If we consider the calculation of weights in GSCA, the estimation occurs per latent factor (either gene or clinical pathway in this case), so varying parameters to increase the number of components in the weighted sum and the number of components with an effect in each sum have substantial effects. Some of these factors can be artificially controlled, for example by limiting the number of components to be substantially less than the sample size. This result from the simulation seems to provide a plausible explanation for the difference in the SLIC data results using the imputed (N SNPs=288) and raw (N SNPs=64) data. Including the additional imputed SNPs into the second analysis appears to have watered down the effect and consequently led to type II error.

Typical sample sizes found in genetics research will likely mitigate some of the power issues occurring from the proportion of significant SNPs. The maximum sample size tested in this simulation study, N = 500, was much better powered to detect fewer significant SNPs among large numbers of non-significant SNPs then the smaller N simulations, although the large N sample size would be expected to give relatively low power at the single SNP level. Our focus was to test the robustness of significant associations found using the GSCA method with the more modest samples typically found in some genetic conditions. We found that common recommendations for achieving statistical power are not different from our findings, i.e. larger sample sizes are particularly important if smaller effect sizes are expected, but we make the additional recommendation for researchers to consider the number of potential effects tested either through LD pruning or the selection of previously associated SNPs/LD blocks.

The second simulation tested the regression-based single SNP association method, which is commonplace in larger genome-wide association studies against the GSCA method. The method was tested for its application to small sample studies which typically have smaller numbers of SNPs and, in some cases, smaller effect sizes. The pattern of results was relatively clear cut regarding both effect size and sample size. The statistical power was significantly compromised when small sample size and, or small effect size were present. Comparing relative results between the single SNP method to GSCA for small numbers of SNPs, sample size, and effect size, generally, we found that GSCA had greater power to detect SNP-phenotype associations. The results are not directly comparable as GSCA works at the gene level, but harnesses information at the SNP level to indicate association between gene and phenotype. The multiple comparisons correction is not required at the SNP level in the GSCA method ( Romdhani *et al*., 2015), so we typically detect more associated SNPs, by considering significance at gene level, than the single SNP method without increasing false positives.

Due to the limitations in sample size, it is necessary to recommend that the GSCA approach should only consider confirmatory candidate gene association studies with a moderate number of SNPs and genes rather than as an exploratory technique to search for associations across the genome. However, GSCA offers the potential to explore more complex relations between genes and multiple clinical pathways within a single model framework (see Romdhani *et al*., 2015 for further discussion and simulations).

## Data availability

### Underlying data

Open Science Framework: Generalized Structured Component Analysis in Candidate Gene Association Studies: Applications and limitations. https://doi.org/10.17605/OSF.IO/PCWY3 ( Thompson *et al.*, 2019)

This project contains the following underlying data:

- alldat_SLIC_select.csv (SNP and phenotype data without imputed SNPs - required for GSCA on imputed SLIC data)
- alldat_SLIC_select2.csv (SNP and phenotype data with imputed SNPs - required for GSCA on imputed SLIC data)
- Random_LD_pheno_random_pattern_negatives_01.csv (output from the simulations for the random pattern containing total number of SNPs, number of SNPs with an effect, number of genes, effect size (correlation), statistical power estimate, number of iterations per simulation, number of subjects, phenotype-phenotype correlation)
- SLICcombos.csv (combinations of parameters for all runs in the simulations)
- SLICmergedPROcounttest.csv (SNP and phenotype data without imputed SNPs - required for GSCA on unimputed SLIC data)
- test_combos_regression_plink_rep500.csv (output from each simulation for the SLIC-sampled pattern containing number of SNPs with an effect, total number of SNPs, number of genes, number of subjects, effect size (correlation), phenotype-phenotype correlation, Bonferroni corrected statistical power estimate [SNPs only], Bonferroni corrected statistical power estimate [SNPs*phenotypes])

### Extended data

Open Science Framework: Generalized Structured Component Analysis in Candidate Gene Association Studies: Applications and limitations. https://doi.org/10.17605/OSF.IO/PCWY3 ( Thompson *et al.*, 2019)

This project contains the following extended data:

- GSCA_Extended_data.docx (supplementary tables A1- A7)

Data are available under the terms of the Creative Commons Zero “No rights reserved” data waiver (CC0 1.0 Public domain dedication).

## Software availability

Source code available from: https://github.com/p1981thompson/GSCA_simulation/tree/GSCA_sims

Archived source code at the time of publication: https://zenodo.org/badge/DOI/10.5281/zenodo.4059401.svg ( Bishop & Thompson, 2020)

License: Creative Commons Zero “No rights reserved” data waiver (CC0 1.0 Public domain dedication)
