# Supplementary material for: Generalized Structured Component Analysis in candidate gene association studies: applications and limitations
Source: Wellcome Open Res. 2020 Oct 8;4:142. Originally published 2019 Sep 25. [Version 2] doi: 10.12688/wellcomeopenres.15396.2 (PMC7818107; doi:10.12688/wellcomeopenres.15396.2)
Supplement: Peer review history

# Peer review: Reviewer response for version 2

**DOI:** 10.21956/wellcomeopenres.17983.r41805

**Published:** 2021-02-19

**Authors:** Alejandro Q. Nato, Jr.

**Affiliations:** 1 Department of Biomedical Sciences, Joan C. Edwards School of Medicine, Marshall University, Huntington, WV, USA

Thompson *et al*. present an engaging, well-written article about the application and assessment of Generalized Structured Component Analysis (GSCA) as a suitable SEM alternative method to replicate results of association studies, when candidate variants for a particular complex trait/disease have already been identified.

 I enjoyed reading the manuscript since the authors described their methods clearly and performed appropriate simulations to evaluate the capabilities of GSCA. I have a few minor comments and suggestions below:

1. Briefly state the reason why SNPs were filtered using MAF > 0.03 and LD < 0.8 (at this first occurrence in the manuscript) and how these specific values were determined. (Methods: SNP Selection, paragraph 3, page 5).
2. As early as Figure 5, indicate what the top ribbon, right ribbon, and x-axis represent. (Results: Part 2, paragraph 2, page 10).
3. Elaborate further why the apparent differences between the random and SLIC-based simulations are likely due to the LD structure differences. (Results; Part 2, paragraph 6, page 10).
4. Briefly mention why testing at effect size of 0.5 was not needed or performed. (Discussion, paragraph 6, page 13).
5. Mention near end of the Discussion whether or not this method can be potentially applied to results from family-based association analyses.
6. Very minimal typographical errors [“conducted directly” should be “conducted on directly” (Results Part 1: SLICdata…, first sentence, page 7); “upto” should be “up to” in Figure 2 (page 7); “leading to a common” should be “leading to common” (Discussion, paragraph 3, last sentence, page 12)].

Is the work clearly and accurately presented and does it cite the current literature?

Yes

If applicable, is the statistical analysis and its interpretation appropriate?

Yes

Are all the source data underlying the results available to ensure full reproducibility?

Yes

Is the study design appropriate and is the work technically sound?

Yes

Are the conclusions drawn adequately supported by the results?

Yes

Are sufficient details of methods and analysis provided to allow replication by others?

Yes

Reviewer Expertise:

Computational and statistical genetics.

I confirm that I have read this submission and believe that I have an appropriate level of expertise to confirm that it is of an acceptable scientific standard.

# Peer review: Reviewer response for version 2

**DOI:** 10.21956/wellcomeopenres.17983.r41803

**Published:** 2021-01-20

**Authors:** Sara Mascheretti

**Affiliations:** 1 Child Psychopathology Unit, Scientific Institute, Istituto di Ricovero e Cura a Carattere Scientifico (IRCCS) Eugenio Medea, Bosisio Parini, Italy

In this manuscript, Thompson and colleagues propose GSCA as a useful method for replication studies, when candidate SNPs have been identified.

 This is a really interesting approach as it represents a well-time contribution to the scientific debate on the dichotomy between candidate gene studies and GWAS by providing evidence about the usefulness of studies based on well-established candidate genes in assessing the functional link between genes and behavior. Moreover, this article provide evidence about the usefulness of using composite scores (i.e. a weighted sum of SNPs with and without an effect) for replication or validation of gene-phenotype associations for which inconsistent results already exist.

 I do not have any major comments/remarks to make.

 The presentation is on the whole clear and the results interesting. The introduction concisely explains the significance and context of this study, and clearly explains the study goals and hypotheses are explained clearly. The methods are well described and fit the study purpose. The results are concise, clear, and supported by appropriate figures and tables. The interpretations of the results are adequate and presented within the broader research context.

Is the work clearly and accurately presented and does it cite the current literature?

Yes

If applicable, is the statistical analysis and its interpretation appropriate?

Yes

Are all the source data underlying the results available to ensure full reproducibility?

Yes

Is the study design appropriate and is the work technically sound?

Yes

Are the conclusions drawn adequately supported by the results?

Yes

Are sufficient details of methods and analysis provided to allow replication by others?

Yes

Reviewer Expertise:

Investigation of the genetic, brain and cognitive bases of reading (dis)ability.

I confirm that I have read this submission and believe that I have an appropriate level of expertise to confirm that it is of an acceptable scientific standard.

# Peer review: Reviewer response for version 2

**DOI:** 10.21956/wellcomeopenres.17983.r40788

**Published:** 2020-10-09

**Authors:** Jan C. Frijters

**Affiliations:** 1 Department of Child and Youth Studies, Brock University, St. Catharines, Canada

No further comments to make. The authors have addressed all of my comments/concerns. The paper will continue to make an important contribution!

Is the work clearly and accurately presented and does it cite the current literature?

Yes

If applicable, is the statistical analysis and its interpretation appropriate?

Yes

Are all the source data underlying the results available to ensure full reproducibility?

Yes

Is the study design appropriate and is the work technically sound?

Yes

Are the conclusions drawn adequately supported by the results?

Partly

Are sufficient details of methods and analysis provided to allow replication by others?

Yes

Reviewer Expertise:

Generally child development and in particular developmental learning disorders, with a focus on reading and reading-related comorbidity. Recent work has focused on the brain and genetic bases of reading disability, ADHD and to a lesser extent DLD.

I confirm that I have read this submission and believe that I have an appropriate level of expertise to confirm that it is of an acceptable scientific standard.

# Peer review: Reviewer response for version 1

**DOI:** 10.21956/wellcomeopenres.16826.r36794

**Published:** 2020-06-16

**Authors:** Jan C. Frijters

**Affiliations:** 1 Department of Child and Youth Studies, Brock University, St. Catharines, Canada

"Generalized Structured Component Analysis in candidate gene association studies: Applications and limitations" reports on the performance of a SEM alternative for establishing and characterizing gene-phenotype associations. Generalized structured component analysis promises to remediate some of the weaknesses of univariate/brute force genetic analyses (i.e., the conundrum of multiple testing, etc.). The authors cogently summarize the dichotomy in standard methods, with candidate gene studies' tendency to miss important effects in different locations on the genome on the one hand and traditional GWAS' tendency to miss small effects on the other hand. 

 A key gateway to the benefits of GSCA is the selection of SNPs putatively involved in a pathway. The example cited from some of the authors' own work is the identification of a null finding in +X/+Y chromosome cases, a rare clinical population. This is one place the introduction of the article could be improved. On what basis are pathways identified? What are the methods by which the search space is constrained? There is also some overlapping use of the term pathway/paths/path. In some cases, 'paths' refer to model-based estimation constraints and in some cases 'paths' refers to clusters of genetic effects, or neurodevelopmental processes. A clearer separation and/or clarification of where this term overlaps or is unique to the specific case would help clarify the arguments.

 These issues become clearer by implication (i.e., all snps within a gene previously identified as having association with a phenotype) as the methodology is explained and the computational example is worked out, but some telegraphing of these issues would enhance the introduction. On page 5 in the methodology, details of pruning CNTNAP2 from 554 down to the constrained snps that were modeled is an example that should be explicated. This description seems to imply that substantial past investigations/knowledge base are required to optimize inputs to the GSCA models. Perhaps this point should be made more explicit, with the boundary conditions and/or specific requirements specified for achieving optimal input to a GSCA.

 The main conclusion of the article is that GSCA can be profitably used for replication of previously identified SNPs. The authors do not clarify how GSCA would be an improvement over a univariate method of replication with a less strict correction for multiple testing. I suspect there may be an advantage, and to a certain extend this advantage is implicit to the arguments of the paper (i.e., see statements under Results, first paragraph: "whether GSCA is less prone to identify spurious associations...". In addition, the results section entitled "Alternative approach simulations" partially addresses this in comparing the best practice PLINK analysis in simulation to GSCA in simulation. However, in replications, multiple repeated linear SNP/phenotype correlations are typically not performed with 0.05/(N SNPS x N phenotypes) as the correction factor - the usage for which GSCA could substitute/improve on that the authors ultimately recommend.

 In the methodology description, the WISC Perceptual Reasoning Index was a good selection for an estimate of IQ in a SLI population, given the strong clinical literature detailing the relative independence of these WISC subtests, taken together, from verbal reasoning.

 The following sentence needs to be revised for clarity, "Unlike structural equation modelling (SEM), GSCA does not have the same assumptions on distributions of variables to ensure multivariate normality." Perhaps, "Unlike structural equation modelling (SEM), GSCA does not necessarily require univariate normality to ensure multivariate normality; however, multivariate normality remains an important requirement in GSCA models.", though I will confess to a lack of knowledge of GSCA models and am unsure if the latter portion of my recommended sentence is correct.

 Perhaps the greatest limitation (and perhaps, fortunately, resulting in a conservative test of the best elements of GSCA) is the nature of the phenotype sample itself. The underlying assumption of the language phenotype involved is the relatively extreme end of an ability that is well-distributed throughout the whole population. The phenotypic assessments (i.e., CELF, WISC, etc.) are all predicated on this model of development for the skills/abilities/functions assessed by the measures. The present study, because of an interest in the clinical end of the language spectrum, is based on a truncated distribution of language skill/development. The genetic assessment is not similarly truncated. In the absence of a non-linear gene-phenotype relationship, this should result in a remarkably underpowered analysis. The in non-linear case, this may enhance or reduce power, depending on the shape of the relationship. Some discussion of this is warranted, especially since the overall argument of the authors is that detecting multiple small effects is an area that needs improvement in current association studies.

 In sum, this manuscript presents GSCA as an alternative to replication or validation of gene-phenotype associations for which some evidence already exists. The degree of associative evidence needed to make the journey into GSCA worth the effort needs to be better specified. Similarly, clarification of a few terminology issues could enhance the overall message. One potentially beneficial effect of an approach such as GSCA could be the aggregation of multiple known snp-phenotype associations in the same direction into an estimate of the composite weight of association, as in the model paths represented in Figure 4. There are several compelling uses for such an estimated value, including aggregation of estimates of gene effect for meta-analyses, or generating risk weights on a per participant basis for classification or prediction studies. While speculative, these value-add components of GSCA could be discussed.

Is the work clearly and accurately presented and does it cite the current literature?

Yes

If applicable, is the statistical analysis and its interpretation appropriate?

Yes

Are all the source data underlying the results available to ensure full reproducibility?

Yes

Is the study design appropriate and is the work technically sound?

Yes

Are the conclusions drawn adequately supported by the results?

Partly

Are sufficient details of methods and analysis provided to allow replication by others?

Yes

Reviewer Expertise:

Generally child development and in particular developmental learning disorders, with a focus on reading and reading-related comorbidity. Recent work has focused on the brain and genetic bases of reading disability, ADHD and to a lesser extent DLD.

I confirm that I have read this submission and believe that I have an appropriate level of expertise to confirm that it is of an acceptable scientific standard, however I have significant reservations, as outlined above.

# Response

**Published:** 2020-09-30

**Authors:** Paul Thompson

**Affiliations:** University of Oxford, UK

**Reviewer 1 Rebuttal: Generalized Structured Component Analysis in candidate gene association studies: applications and limitations**

**[version 1; peer review: 1 approved with reservations]**

**Jan C. Frijters**, Department of Child and Youth Studies, Brock University, St. Catharines, Canada 

**"Generalized Structured Component Analysis in candidate gene association studies: Applications and limitations" reports on the performance of a SEM alternative for establishing and characterizing gene-phenotype associations. Generalized structured component analysis promises to remediate some of the weaknesses of univariate/brute force genetic analyses (i.e., the conundrum of multiple testing, etc.). The authors cogently summarize the dichotomy in standard methods, with candidate gene studies' tendency to miss important effects in different locations on the genome on the one hand and traditional GWAS' tendency to miss small effects on the other hand.**

**A key gateway to the benefits of GSCA is the selection of SNPs putatively involved in a pathway. The example cited from some of the authors' own work is the identification of a null finding in +X/+Y chromosome cases, a rare clinical population. This is one place the introduction of the article could be improved. On what basis are pathways identified? What are the methods by which the search space is constrained? There is also some overlapping use of the term pathway/paths/path. In some cases, 'paths' refer to model-based estimation constraints and in some cases 'paths' refers to clusters of genetic effects, or neurodevelopmental processes. A clearer separation and/or clarification of where this term overlaps or is unique to the specific case would help clarify the arguments.**

Response:

**“On what basis are pathways identified?”**

Pathways were based on specific hypothesis of structure of the pathways based on knowledge of the literature, specifically familiarity with existing identified genes/SNPs and aetiology of specific language impairment (or DLD).

This is explained in the section on Candidate Gene Selection, just below Table 1.  SNPs are grouped into pathways according to which gene they belong to, and the four genes were selected on the basis of prior literature, with two cases where an association was expected, and two where it was not.

**“What are the methods by which the search space is constrained?”** 

The search space was constrained to SNPs and genes that had been previously identified. This reduced the search space considerably and tries to demonstrate the replicability of previous findings. Future use of GSCA could consider using identified SNPs from overlapping fields or if the researcher has specific hypotheses about a set of SNPs. As we emphasize in our discussion, the use of GSCA is not well suited for mass exploratory analysis. In areas such as neurodevelopmental disorders, however, one typically finds that there are a few genetic associations that have replicated in at least two samples, but where doubt remains about the robustness of association. It is this kind of situation where we suggest GSCA could be useful in future.

**“There is also some overlapping use of the term pathway/paths/path. In some cases, 'paths' refer to model-based estimation constraints and in some cases 'paths' refers to clusters of genetic effects, or neurodevelopmental processes. A clearer separation and/or clarification of where this term overlaps or is unique to the specific case would help clarify the arguments.”**

**These issues become clearer by implication (i.e., all snps within a gene previously identified as having association with a phenotype) as the methodology is explained and the computational example is worked out, but some telegraphing of these issues would enhance the introduction.**

Response:

We agree with the reviewer and have substituted the following paragraph in the introduction to address this. See lines…. 

“We compared the findings of the GSCA with conventional single SNP analysis, for four groups of SNPs, identified on the basis of prior literature. The first two groups were SNPs located on genes that had previously been shown to be associated with quantitative speech and language phenotypes in the full SLIC data set (Study Part 1). Here we predicted that GSCA should confirm the association. These two groups were contrasted with SNPs from two other genes that acted as negative controls, in that we predicted they would not show association with DLD phenotypes.”

**On page 5 in the methodology, details of pruning CNTNAP2 from 554 down to the constrained snps that were modeled is an example that should be explicated. This description seems to imply that substantial past investigations/knowledge base are required to optimize inputs to the GSCA models. Perhaps this point should be made more explicit, with the boundary conditions and/or specific requirements specified for achieving optimal input to a GSCA.**

Response: 

The pruning described simply involved the restriction of window size within the candidate gene and was performed to reduce the number of SNPs included in the analyses. Whilst this required some prior knowledge from the reviewed literature base, no specialist knowledge is required to perform this pruning step. We have now made this clear in the methods (page 5)

As GSCA is primarily used as a confirmatory hypothesis approach for prespecified genetic pathways, there is some requirement on the user to have an amount of prior information to narrow the field of SNPs entered into the model. In this paper, we use previously reported associations and regions of interest to select SNPs to test within a hypothesised pattern of clinical and genetic pathways. 

*“CNTNAP2* and *ATP2C2* are large genes containing many SNPs; therefore, we filtered these two genes to include only those regions of association that had been previously published.”

We also state in the discussion:

“we make the additional recommendation for researchers to consider the number of potential effects tested either through LD pruning or the selection of previously associated SNPs/LD blocks.”

We explicitly state that the method is not well suited to mass-exploratory analysis, but as a means to robustly detect complex pathways that may be missed or falsely identified using a more conventional univariate approach.

**The main conclusion of the article is that GSCA can be profitably used for replication of previously identified SNPs. The authors do not clarify how GSCA would be an improvement over a univariate method of replication with a less strict correction for multiple testing. I suspect there may be an advantage, and to a certain extend this advantage is implicit to the arguments of the paper (i.e., see statements under Results, first paragraph: "whether GSCA is less prone to identify spurious associations...". In addition, the results section entitled "Alternative approach simulations" partially addresses this in comparing the best practice PLINK analysis in simulation to GSCA in simulation. However, in replications, multiple repeated linear SNP/phenotype correlations are typically not performed with 0.05/(N SNPS x N phenotypes) as the correction factor - the usage for which GSCA could substitute/improve on that the authors ultimately recommend.**

Response:

PLINK offers a range of multiple testing correction options including Bonferroni - https://zzz.bwh.harvard.edu/plink/anal.shtml#adjust 

For a restricted SNP set, we would typically use Bonferroni. This is simple, everyone understands it and accepts that it is usually over-conservative. However, for a larger SNP set, we consider the permutation options provided within PLINK (https://zzz.bwh.harvard.edu/plink/perm.shtml). This allows for the dataset in hand and deviations from normality for continuous phenotypes. But permutation still considers a single SNP at a time – you should still apply multiple testing

We have considered the reviewer’s point and have changed the reported PLINK correction to the FDR (Benjamini-Hochberg) which is less conservative than the Bonferroni to provide a fairer comparison.

Figure 7 has been updated to reflect the change to the power curves for the FDR. It can be seen that the PLINK method retains more power than previous version of reported results but there is still a substantial benefit to the power when using GSCA for small samples. This is the main conclusion from the paper and is stated in the discussion. As there is substantially more power, we are less prone to identify spurious associations.

**In the methodology description, the WISC Perceptual Reasoning Index was a good selection for an estimate of IQ in a SLI population, given the strong clinical literature detailing the relative independence of these WISC subtests, taken together, from verbal reasoning.**

Response:

We thank the reviewer for this comment.

**The following sentence needs to be revised for clarity, "Unlike structural equation modelling (SEM), GSCA does not have the same assumptions on distributions of variables to ensure multivariate normality." Perhaps, "Unlike structural equation modelling (SEM), GSCA does not necessarily require univariate normality to ensure multivariate normality; however, multivariate normality remains an important requirement in GSCA models.", though I will confess to a lack of knowledge of GSCA models and am unsure if the latter portion of my recommended sentence is correct.**

Response:

We agree that the reviewer’s suggestion for re-wording is an improvement and have implemented. 

**Perhaps the greatest limitation (and perhaps, fortunately, resulting in a conservative test of the best elements of GSCA) is the nature of the phenotype sample itself. The underlying assumption of the language phenotype involved is the relatively extreme end of an ability that is well-distributed throughout the whole population. The phenotypic assessments (i.e., CELF, WISC, etc.) are all predicated on this model of development for the skills/abilities/functions assessed by the measures. The present study, because of an interest in the clinical end of the language spectrum, is based on a truncated distribution of language skill/development. The genetic assessment is not similarly truncated. In the absence of a non-linear gene-phenotype relationship, this should result in a remarkably underpowered analysis. The in non-linear case, this may enhance or reduce power, depending on the shape of the relationship. Some discussion of this is warranted, especially since the overall argument of the authors is that detecting multiple small effects is an area that needs improvement in current association studies.**

Response:

This is a reasonable concern, given the fact that the sample came from the SLIC cohort. However, the way that DLD is defined means that a child may obtain good scores on some language measures but not on others. Table 1 shows the range of scores for the three phenotype measures; while the range is restricted for the Expressive Language phenotype, it covers a very broad span for the other two phenotypes. 

**In sum, this manuscript presents GSCA as an alternative to replication or validation of gene-phenotype associations for which some evidence already exists. The degree of associative evidence needed to make the journey into GSCA worth the effort needs to be better specified. Similarly, clarification of a few terminology issues could enhance the overall message. One potentially beneficial effect of an approach such as GSCA could be the aggregation of multiple known snp-phenotype associations in the same direction into an estimate of the composite weight of association, as in the model paths represented in Figure 4. There are several compelling uses for such an estimated value, including aggregation of estimates of gene effect for meta-analyses, or generating risk weights on a per participant basis for classification or prediction studies. While speculative, these value-add components of GSCA could be discussed.**

Response:

We thank the reviewer for their constructive comments and recommendations. These additions improve the readability and clarity. We have added a section in the discussion based on the reviewer’s suggestion, although we only briefly discuss the potential to explore relationships between multiple pathways and genes. This topic is fully discussed in Romdhani et al. (2015) within their simulations.  

· **Is the work clearly and accurately presented and does it cite the current literature?**

Yes

· **Is the study design appropriate and is the work technically sound?**

Yes

· **Are sufficient details of methods and analysis provided to allow replication by others?**

Yes

· **If applicable, is the statistical analysis and its interpretation appropriate?**

Yes

· **Are all the source data underlying the results available to ensure full reproducibility?**

Yes

· **Are the conclusions drawn adequately supported by the results?**

Partly

Competing Interests

*No competing interests were disclosed.*
